# Assessing Food Access, Exercise, and Dietary History among Older African American Parishioners During the COVID-19 Pandemic (C-FED Study): Design, Opportunities, Challenges, and Lessons Learned

**DOI:** 10.1007/s40615-023-01657-8

**Published:** 2023-06-19

**Authors:** Lucy W. Kibe, Adaobi Bosah, Katrina M. Schrode, Yufu Kuo, Magda Shaheen, Edward Adinkra, Humberto Sanchez, Mohsen Bazargan

**Affiliations:** 1 Physician Assistant Program, Charles R. Drew University of Medicine and Science, 1731 E. 120Th St., Los Angeles, CA 90059, USA; 2 Department of Psychiatry, Charles R. Drew University of Medicine and Science, Los Angeles, CA 90059, USA; 3 Department of Internal Medicine, Charles R. Drew University of Medicine and Science, Los Angeles, CA 90059, USA; 4 Department of Family Medicine, Charles R. Drew University of Medicine and Science, Los Angeles, CA 90059, USA; 5 Office of Research, Charles R. Drew University of Medicine and Science, Los Angeles, CA 90059, USA; 6 Department of Family Medicine, University of California Los Angeles (UCLA), Los Angeles, CA 90059, USA

**Keywords:** African American, Older Adult, COVID-19, Diet, Exercise, Food access

## Abstract

**Objectives:**

Unhealthy diets and inadequate exercise are associated with chronic health conditions and excess mortality. Older African Americans do not meet dietary and exercise guidelines, and this may have worsened during the COVID-19 pandemic due to individual and environmental factors, including food insecurity. Studies evaluating these dynamics are essential for developing interventions. This narrative details a study protocol and data collection experiences during the pandemic.

**Methods:**

Participants > 55 years African American old completed detailed food frequency, exercise, and food access questionnaires between October 2020 and July 2021. Observations of the study administrators (authors of this manuscript) for the duration of the study are presented. Details on the study design and reflections on the opportunities, challenges, and lessons learned are summarized. Future manuscripts will report data analysis of study findings.

**Results:**

A total of 123 older African American adults participated in the study, and 118 (70% female) completed all three questionnaires. More than 50% of the participants had at least two primary chronic conditions. About 85% were fully vaccinated against COVID-19. Applying community-based participatory approaches, leveraging partnerships, and exercising flexibility approaches were pivotal to successfully implementing the study protocol.

**Conclusions:**

Despite challenges related to the COVID-19 pandemic, detailed data on older African American adults’ diet and exercise habits were obtained. Our study design and experiences will benefit future researchers. More importantly, results from our study will inform interventions and policies aimed at minimizing consequences associated with poor diet and exercise habits during the pandemic among this vulnerable population.

## Background

African Americans (AA) and older adults have suffered from higher infection rates, hospitalization, and death during the COVID-19 pandemic [1, 2]. In June 2022, the CDC reported that AA are 1.1 × more likely to get infected, 2.3 × more likely to be hospitalized, and 1.7 × more likely to die from COVID-19 compared to Whites [3]. Although all older adults experienced higher infection rates than younger adults, AA over the age of 50 were more likely to die compared to Whites of the same age [1]. This disparity is partially attributed to the high prevalence of chronic conditions associated with COVID-19 severity among older AAs, including obesity, hypertension, and diabetes [2, 4]. These chronic conditions are in turn, associated with lifestyle habits, including exercise and diet.

It is well established that unhealthy diets and inadequate exercise are associated with the prevalence of many chronic conditions and contribute to poor outcomes, including death [5, 6]. This is particularly important among older adults, whose dietary habits and exercise may be affected by age-related factors such as changes in nutritional needs, chronic diseases, physiological changes, physical and cognitive limitations, social isolation, and financial constraints [7, 8]. However, many older AA are less likely to meet recommended dietary guidelines for a healthy diet [9–16] and exercise [6, 9].

While consequent to the COVID-19 pandemic, a worsening of unhealthy dietary habits, including increased consumption of junk food, low fruit and vegetable intake, and sedentary habits has been reported overall [17], older adults may have been hit worse [18–20]. A systemic review examining the effect of the COVID-19 lockdown on adults over age 50 postulated that socioeconomic status and food availability may be responsible for dietary changes, reduced physical activity, and an increase in sedentary lifestyle [18]. Reports earlier in the pandemic indicated a substantial increase in households reporting new or increased food insecurity [21, 22]. Food insecurity was reported to be worse among African Americans [23] and among older adults [24]. One study that examined the impact of COVID-19 among a low-income, predominantly AA cohort showed approximately 80% increase in food insecurity [25]. Despite an increase in Supplemental Nutritional Assistant Program (SNAP) during the COVID pandemic, enrolled families still reported food insecurity [26]. Increased food insecurity during the pandemic is likely a consequence of safety measures resulting in reduced availability of food, decreased purchasing power, restrictions of supermarket and exercise areas, and fear of being in public places.

Because inadequate exercise and unhealthy diet may cause long-term challenges on disease and healthy aging beyond the COVID-19 pandemic, we examined exercise habits, dietary history, and food access among older AA adults during the pandemic. This manuscript is a reflective narrative describing the collaborative process and approach utilized by a university and local partnering churches for this study. This report documents the opportunities, challenges, and lessons learnt to inform future strategies for similar studies.

## Methods

The Food Insecurity, Exercise, and Dietary history (C-FED) study was an “add-on” to a larger COVID-19 longitudinal intervention study. We refer to the latter as the “Parent Study.”

### Parent Study Design

The design of the Parent Study will be detailed in another manuscript. We briefly describe the study here. The Parent study aims were to mitigate the unfavorable impact of COVID-19 on the management of chronic health conditions, as well as reduce healthcare avoidance behaviors and psychological distress among older African Americans within and around Los Angeles County. To achieve its aims, Charles R. Drew University of Medicine and Science formed a partnership with 10 African American churches. Thirty (30) trusted members and stakeholders were recruited from the ten (10) African American churches and trained by the study team to serve as health ambassadors and deliver the intervention. One key church leader was officially appointed as a community faculty at the university to facilitate the study and promote the community voice in this partnership. The Parent Study began data collection in November 2020.

During the development of the Parent Study, the Principal Investigators (PI) identified the need to expand questionnaire items evaluating food insecurity, exercise, and dietary intake. However, a thorough evaluation of these areas of study were outside the scope of the Parent Study. Therefore, additional funding and expertise was sought to expand and complement the Parent Study in these areas. Hence, the C-FED joined in October 2021. All participants in the C-FED study were enrolled in the Parent Study. Participants of the Parent Study were considered eligible if they were African American adults (all genders) aged 65 years and older or 55–64 years old with at least one chronic medical condition. Participants were excluded if they were institutionalized, had a cognitive deficit (identified by an abridged version of a mini-mental instrument), or unable to speak and/or read English. Informed consent was obtained from all individual participants included in the study.

### C-FED Study Design

The C-FED study was a cross-sectional assessment of food insecurity, exercise, and dietary intake during the COVID-19 pandemic. Data collection volunteers comprising current and recent undergraduate and graduate students were recruited through an announcement in the Charles R. Drew University of Medicine and Science Student Services Weekly Newsletter. These volunteers received intensive training on the administration of the C-FED study questionnaires. The training involved questionnaire administration training, answering the questionnaire (as participants would), and observation of the volunteer administering the questionnaires and receiving feedback. The C-FED study team met bi-weekly to review progress and receive feedback on data collection and study logistics.

#### Data collection

Survey data were collected between October 2021 and July 2022. During this period, there were more than 1.4 million reported cases of COVID-19 and more than 26,106 deaths in Los Angeles County alone [27]. Due to the pandemic restrictions, three modes of data collection were utilized based on participant preference: self-administration (online link sent by email), telephone, or in-person interviews. All in-person interviews were conducted during organized field trips in collaboration with church leaders and health ambassadors and held at one of the churches. A total of four in-person interview sessions were conducted at two different churches, as follows: two in December 2021, one in March 2022, and one in July 2022. Because data were entered directly into cloud-based databases, internet access was needed. The study provided external internet Hotspots when internet access was not available at the churches. The study was approved by the Charles R. Drew University of Medicine and Science institutional review board.

#### Study Measurements

##### Food Access

Food access was measured using a brief survey adapted from www.phenxtoolkit.org. The survey has two sections: food environment and food insecurity.

Food environment was measured by access to healthy food in the neighborhood (defined as within a 20-minwalk or 1 mile from home) by three questions. Q1. “the fresh fruits and vegetables in my neighborhood are of high quality”; Q2. “a large section of fresh fruits and vegetables is available in my neighborhood”; and Q3. “a large selection of low-fat products is available in my neighborhood.” Response options were the following: “strongly agree/agree/neither agree nor disagree, disagree/strongly disagree.”

Food insecurity was measured using the USDA U.S. Household Food Security Survey: 6-item short form. The survey instrument consists of the following six questions about participants’ food situation. In the last 12 months:

Q1. “The food that (I/we) bought just didn’t last and (I/we) didn’t have money to get more”; Q2. “(I/we) could not afford to eat balanced meals.” Response options to Q1 and Q2 were the following: *often true/sometimes true/never true/don’t know/ refused*. Q3. “in the last past 12 months, did you or (other adults in your household) ever cut the size of your meals or skip meals because there wasn’t enough money for food?” Response options to Q3 were: *yes/ no/don’t know/refused*.

Q4. “In the last 12 months, did you ever eat less than you felt you should because there wasn’t enough money to buy food? and Q5. “In the last 12 months, were you ever hungry but didn’t eat because you couldn’t afford enough food?” Response options to Q4 and Q5 were the following: *yes/ no/don’t know/refused*. Additionally, if any of Q1, Q2 or Q3 were answered affirmatively (i.e., if either Q1 or Q2 were “often true” or “sometimes true” or Q3 was “yes”), a follow-up question was asked: Q3a. “How often did this happen? Response options to Q3a were the following: *almost every month/some months but not every month/ only 1 or 2 months*.

##### Exercise

Physical activity was assessed using the Yale Physical Activity Questionnaire (YPAS) [28]. This survey was developed to determine the type, amount, and pattern of physical activity/exercise in older adults. The YPAS survey has been validated among older African American and Spanish-speaking participants [29, 30]. It is composed of two sections — (a) the amount of various physical activities/exercise performed during a typical week in the past month and (b) activities performed in the past month. The total metabolic equivalent of task (MET) was calculated based on activity, frequency, intensity, and duration.

##### Dietary History

The Diet History Questionnaire (DHQ) III was used for data collection and analysis of dietary intake. This instrument was created by the National Cancer Institute (NCI) and is freely available on the web [31, 32]. Available in English and Spanish languages, it has been validated using 24-h recalls from the National Health and Nutrition Examination Survey (NHANES) and biomarker studies [33]. A demo of the questionnaire is available at https://www.dhq3.org/study/demo/.

The DHQ III comprises 135 food and beverage items and 26 dietary supplements. Participants responded to questions on the type, frequency, and serving sizes of foods, beverages, and dietary supplements consumed within the preceding 12-month period. In addition to separate scores for several nutrients and food groups, an overall score of food quality (the Healthy Eating Index-2015: HEI) can be computed. Because of additional questions embedded in the questionnaire, up to 263 foods and beverages can be assigned in the nutrient and food group database.

The DHQ III nutrient and food group values are derived from USDA’s Food and Nutrient Databases for Dietary Studies, USDA’s Food Pattern Equivalents Databases, and the University of Minnesota’s Nutrient Database for Research. The NCI gives detailed analytical instructions and coding to analyze the DHQ III.

A unique account with a dedicated ID number was established for each participant. Participants who chose to complete the DHQ on their own were reminded that the link could only be used once and was not to be shared with others. Detailed individualized results, compared with the USDA guidelines, were produced for each participant. However, to reduce bias, these results were not made available to the participants during the data collection phase.

##### Sociodemographic and Other Variables

Age and gender were self-reported by the participants. Other sociodemographic variables reported on the Parent Study survey instrument included the following: race, level of education, marital status, and annual household income level. The Parent Study survey instrument included the following health and COVID-19-related variables: (1) baseline health status, (2) healthcare utilization including emergency department use and hospital admissions, (3) COVID-19, influenza, and pneumonia precautions for risk reduction, (4) knowledge and attitudes towards vaccinations, (5) accessibility, acceptability, and affordability of vaccination, (6) vaccination completion and adherence to schedule.

#### Dissemination of DHQ III Survey Results

In collaboration with the church leadership, the study team hosted a gala at the completion of the study. At this event, participants received printouts of their individualized DHQ III results. The study PI gave a presentation on healthy eating guidelines (Myplate for older adults [34]). Step-by-step, the participants were guided to review and self-reflect on their DHQ III results during the presentation. Participants were also encouraged to discuss their results with their healthcare providers. A healthy meal was served during the gala to model healthy eating.

## Results

### Participant Recruitment and Characteristics

Of the 133 participants enrolled in the Parent Study, 123 also participated in the C-FED study. Most of the surveys (79 DHQ III, and 76 YPAS and food access surveys) were completed through telephone interviews. Links sent by email resulted in 20 DHQ III and 28 YPAS and food security surveys. Finally, 19 DHQ III, YPAS, and food security surveys were completed in-person. The time to complete the telephone/in-person interviews for each participant was between 45 and 85 min.

**Fig. 1:**
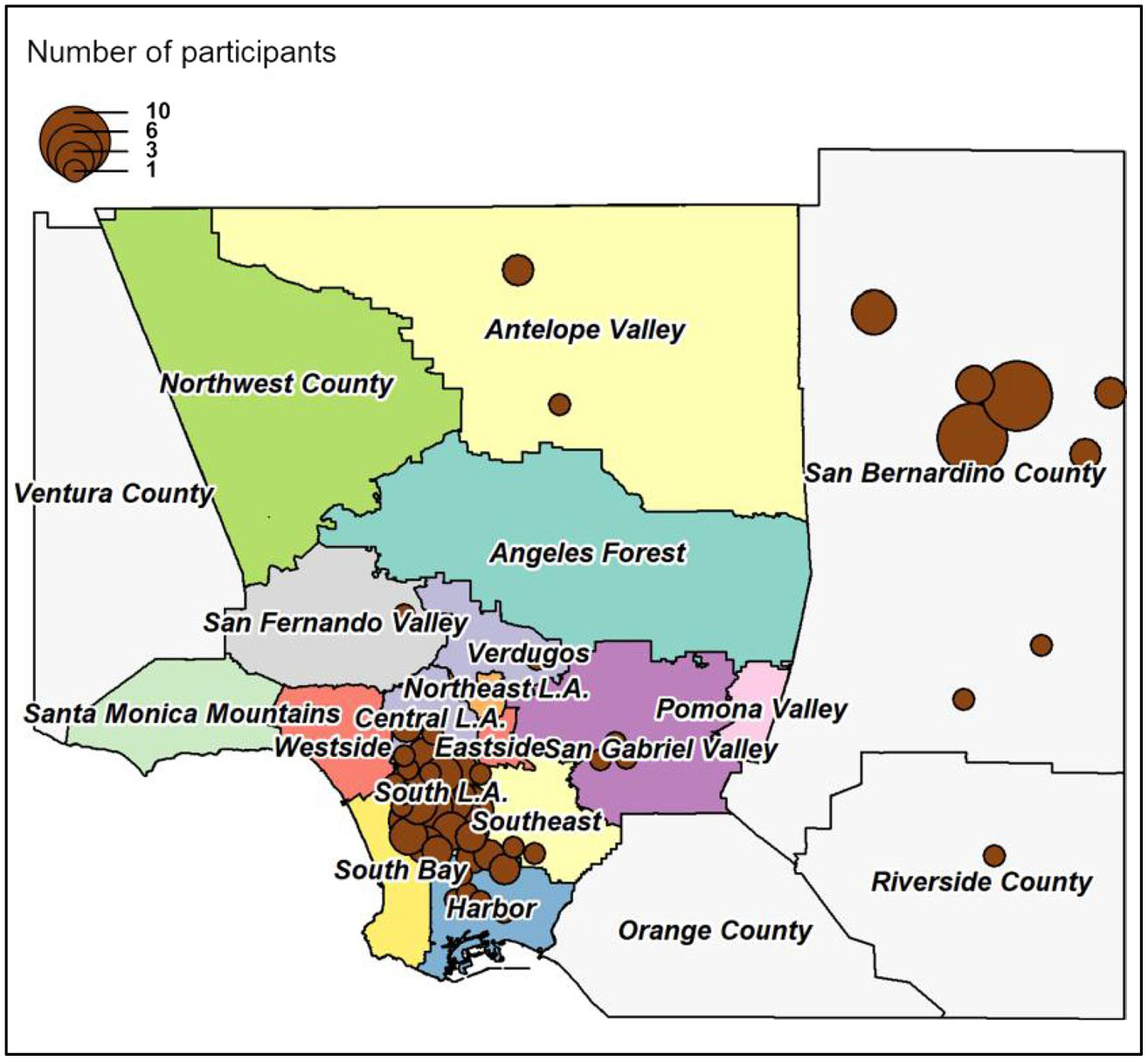
Distribution of study participants by location [53]

Table 1 shows participant enrollment in the C-FED study. About 89% of parent study participants successfully completed all C-FED study surveys. Characteristics of eligible enrolled C-FED participants are shown in Table 2. Approximately 70% of the participants were female. Both males and females were well distributed within age ranges. Participants ranged in education level from less than a high school diploma to completion of graduate or professional degrees. Most participants were not married or living with a partner (62%). While the majority (65%) rated their physical health as good or better, over half had at least 2 primary chronic conditions. 85% of participants were fully vaccinated against COVID-19, and an additional 6% were partially vaccinated. The study participants were recruited from Los Angeles, San Bernadino, and Riverside counties (Fig. 1).

**Fig. 2:**
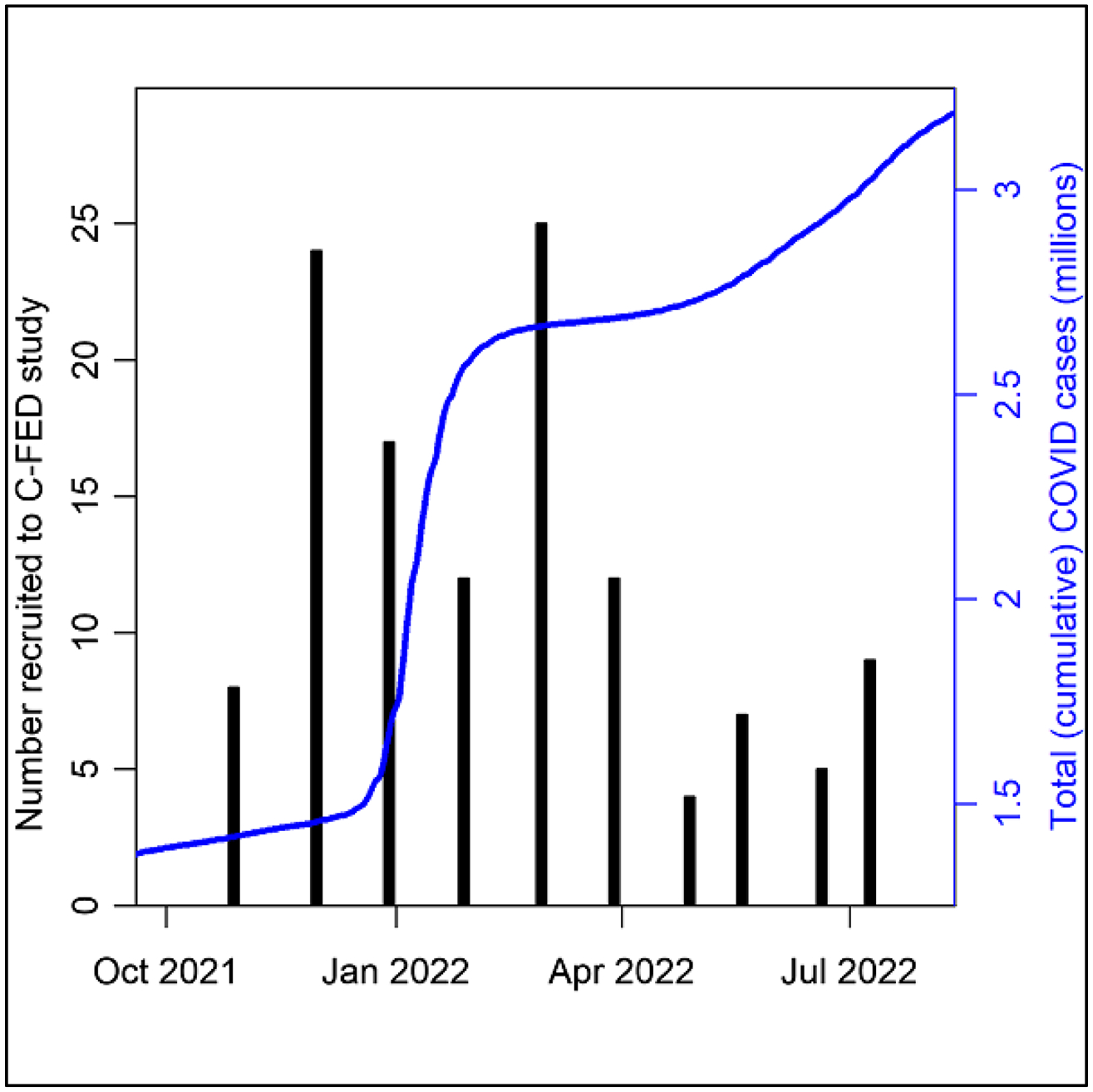
Data collection timeline

Figure 2 plots the cumulative cases of COVID-19 in Los Angeles County during the C-FED study period, along with monthly recruitment into the study. The number of COVID-19 infections continued to rise throughout the duration of the study. The number of COVID-19 cases in Los Angeles County at the beginning of data collection was approximately 1.4 million and rose to approximately 3.3 million in July when data collection ended. Participant recruitment was highest in December 2021 and lowest in May 2022.

## Opportunities and Challenges

Throughout the study period, study administrators (also authors of this manuscript) documented reflections on unique opportunities and challenges associated with collaborations, recruitment, and data collection processes. A compilation of these reflections are summarized in Table 3 and discussed in further detail below.

### A. Opportunities

#### Importance of Study Findings

To our knowledge, this is the first comprehensive study of local food access, diet, and exercise behaviors among older African American adults during COVID-19 in Southern Los Angeles and the surrounding area. Study findings will provide insight for the development of intervention studies aimed at reducing the negative consequences of chronic health conditions associated with poor diet and limited exercise. This is important because the impact of unfavorable lifestyle habits may persist long after the COVID-19 pandemic. Study findings may also apply to other similar populations.

#### Community-Based Participatory Research Approach

Utilizing Community-Based Participatory Research (CBPR) improved the acceptability and relevance of the research study for its participants and their wider communities within and beyond Los Angeles County. This official faculty appointment of a key church leader facilitated a seamless involvement of other church leaders and parishioners in the study design and execution. Many health ambassadors were also church leaders (deacons, pastors, small group leaders, etc.). In the initial planning of the study, the university and churches entered into partnership agreements that clarified the responsibilities and obligations of each partner. These agreements were crucial to providing an anchor for commitment to solve any unforeseen changes within the university or the churches e.g. staff changes, budget cuts, shutdowns, etc.

#### Empowerment and Longevity

Church ambassadors trained by the parent study supported the C-FED study. They were instrumental in enrolling and navigating data collection activities for the C-FED study. These health ambassadors will be engaged in future health interventions.

#### Leveraging Established Research Protocol

By “tagging on” to the Parent Study, the C-FED study was able to leverage resources, increase efficiency, and reduce study costs. Participants had the convenience of signing a combined informed consent form and completing the Parent Study baseline surveys and C-FED study surveys sequentially. Study teams had combined meetings, and shared recruitment, regulatory paperwork, and study report responsibilities.

#### Multiple Data Collection Modalities

Due to COVID-19 safety measures, flexibility of data collection modalities was utilized. This flexibility allowed more participants to be engaged. Participants selecting telephone interviews or self-administration were able to complete the surveys safely at home and without the burden of commuting to the churches.

#### Data Storage

Regardless of collection modality, all survey data was directly stored on cloud-based systems. This approach eliminated the burden of data entry, transcription errors, or data loss. Data was also available in real time for the study team to notice and correct any data collection errors. Extensive training of data collection volunteers and continued monitoring was essential.

#### Teaching and Modeling Healthy Eating

The gala at the end of the study provided an opportunity to inform participants of their individualized dietary patterns and provide guidance on reflective modifications. By sharing a healthy meal, participants were able to interact with one another and the study team, informally discussing healthy eating topics. These discussions are expected to continue between participants and their healthcare providers.

### B. Challenges

#### COVID-19 Restrictions

Some participants preferred in-person interviews. However, due to the COVID-19 restrictions during data collection, and the knowledge that AA adults were at high risk for COVID-19 infection, this was not always possible. Participants without access to online or telephones may have lost the opportunity to participate if they were not available when in-person sessions were organized.

#### Recruitment Challenges

Initially, participants did not clearly understand the scope of the Parent Study and the C-FED study. Although they had signed consent for both studies, some participants were surprised when contacted to complete the C-FED study surveys. To alleviate this misunderstanding, both study teams provided clarity as often as possible. In some instances, telephone numbers provided to the Parent Study changed before the C-FED study team reached the participant. Additionally, some participants were difficult to reach by phone. Both teams worked closely with the church ambassadors to reach these participants. To improve communication, a weekly meeting of all health ambassadors and both study teams was established to obtain feedback, answer questions, and strengthen relationships.

#### Logistical Challenges

While there were many benefits to the C-FED study “tagging along” with the Parent Study, this arrangement diminished the autonomy of the C-FED study staff. Participants had to be first enrolled in the Parent Study. This occasionally delayed the pace of the C-FED study data collection. Frequent communication and sharing recruitment databases reduced these challenges.

#### Participant Technical Challenges

Poor technological savviness, internet inaccessibility/ irregularities, and non-possession of telephones were some of the deterrents to the administration of online and telephone surveys for some of the participants. Some of the participants could only be reached through their relatives. The option of in-person interviews later in the data collection phase alleviated some of these challenges.

#### Recall and Survey Fatigue

As with other survey methodology, recall was a challenge, especially among the much older participants. Additionally, the surveys were long, taking an average of 60–85 min to complete. Some participants with low literacy needed help understanding the questions and therefore took even longer to complete. The data collection volunteers offered an opportunity for the surveys to be done in two sessions if the participant was seamed fatigued.

#### Survey Instruments

To be able to compare findings with previous studies, the C-FED study used standardized, validated surveys. Although this did not happen often, the surveys did not include some foods, or some activities reported by the participants. These were written in and will be reviewed.

#### Interrater Reliability

The study plan included strategies to reduce interrater reliability among data collection volunteers. These volunteers received rigorous training and met weekly to share experiences and receive feedback. Frequent data checks were conducted during the data collection phase. Any anomalies were identified and promptly corrected.

## Discussion

Several studies have reported a global decline in food access, exercise and diet quality associated with the COVID-19 pandemic. However, few studies have focused on underserved older African Americans, a highly marginalized population that is disproportionately burdened by COVID-19 consequences and cardiovascular disease [1, 2, 4]. This paper reports our experience in collecting survey information in this population during the COVID-19 pandemic, including our study design, identified opportunities, challenges, and steps taken to overcome them. We now discuss the lessons learned. Our experience provides valuable information to other researchers engaging in similar work during and post-pandemic.

Faith-based organizations (FBOs) have been shown to be an effective avenue for the advancement and execution of disease prevention and health promotion endeavors among minority populations [35]. In our study, FBOs provided the platform for the CBPR approach, which made a significant impact on the success of the C-FED study. The pastoral leadership provided the internal outlook, which helped to decrease stigma related to study participation, reduced fear, and misconceptions, and encouraged participants who were previously apprehensive about the study. Such noteworthy influence was also reported in an intervention that targeted the prevention of obesity and cancer among African Americans in New York City [36]. The success was attributed to the development of efficient approaches by the project researchers for identifying, recruiting, and partnering with those churches. Initial access into the FBO was through lay leaders, while the intervention’s implementation was dependent on the full support provided by the pastoral leadership. The pastoral leadership also adapted the intervention to fit the needs of their members. A higher participant retention rate (92%) was seen in one FBO [36], where the pastoral leadership provided full support during the 12-week pilot. On the contrary, 62% retention was seen in an FBO where the spiritual leadership approved of the study but did not advocate for the study among the congregation [37].

Both the Parent Study and the C-FED study were a paradigm of community-based research, which leveraged the partnership between the university and various faith-based leaders, who are essential stakeholders in the African American Community. Challenges of CBPR, such as issues of power, privilege, participation, community consent, racial and/or ethnic discrimination, and the role of research in social change, were avoided by involving church leaders in the planning stages, appointing a paid community faculty, and developing a partnership agreement. Others have reported these benefits to the CBPR paradigm [38, 39] and for research involving older AA adults. This provided the opportunity to nurture bonding between researchers and community partners, as well as to discuss complex research principles and share personal challenges and victories. During the pandemic, meetings and trainings were offered using Zoom. As recommended by previous studies [40], it was important for our study to support the health ambassadors to overcome technological challenges by providing laptops and Hotspots for in-person events and remuneration for community partners’ time and effort. Our study went further by appointing a well-respected church leader as a community faculty in our institution. Indigenous elders or leaders of collaborating communities are often valuable resources who can provide researchers with practical strategies that work well in their communities. This provides a platform for meaningful community-based research where academics, clinicians, and community members develop research projects together from the beginning, stemming from the needs of the community.

An important, often overlooked aspect of CBPR is the dissemination of research results back to the community [41, 42]. One study suggests that only 25% of CBPR researchers disseminate research findings back to the community [42]. Dissemination of study findings provides a platform for knowledge translation and exchange, a crucial aspect of research. In our study, disseminating of individualized DHQ III results enabled community members to learn about healthy eating while reviewing and reflecting on their individualized diet histories, asking questions, and receiving feedback.

The collaboration between the Parent Study and the C-FED study teams provided the platform for resource sharing, especially for leveraging established research protocols for the enrollment of subjects by the C-FED study team. Most contemporary biomedical research relies on collaboration. This could mean an equal alliance between two or more members engaged in mutually beneficial research, or the relationship between the collaborators could vary in depth and in the balance of the relationship. Collaborative research provides the opportunity to combine the experiences and expertise of various associates from diverse backgrounds and create a more effective and efficient environment for high-quality results [43, 44] while optimizing time and cost. A good example is research collaboration in pharmaceutical companies, where it facilitates the rapid transition of discoveries from the research bench to hospital clinics [44]. While the diminished autonomy for the C-FED study recruitment and enrollment of study participants affected the pace of data collection, the benefits of collaboration outweighed the drawbacks. Constant communication, uniformity of messages to participants, and the use of shared databases were useful in managing logistics between the collaborating study teams.

Despite the importance of including older adults in research to inform clinical practice, this population continues to be underrepresented [45]. This is especially true among minority populations [46]. Some of the challenges to the success of the recruitment and retention of this population in clinical research include the following: multiple health concerns, leading to frequent hospitalization; social and cultural barriers, including immigration and acculturation complexities, language, and literacy complexities; impaired capacity to provide informed consent; mistrust of research; and poor attitude toward health promotion [38, 45, 46]. Research shows that these barriers and challenges can be addressed and overcome through the following strategies: early and in-depth planning; reducing exclusion criteria; securing cooperation from interested parties; incorporation of advisory boards; timely screening identification and approach of eligible patients; as well as carefully reviewing the benefit-risk ratio for appropriateness [38, 43, 45, 46]. Prior planning allowed our study to incorporate these best practices. Additionally, fewer older men than women participate in research-based health promotion programs. In a study conducted by the Healthy Aging Regional Collaborative of South Florida aimed at gaining insight into the barriers to recruiting and engaging older men in evidence-based health promotion programs, 78% of program coordinators identified the perception of health promotion programs as feminine as a barrier [39]. Over 90% of these program coordinators reported that program advertisements featuring men would increase male participation. Our study adapted these strategies by encouraging health ambassadors and enrolled participants to invite all eligible church members to join the study, regardless of their gender.

Because our data collection, by design, was during the COVID-19 pandemic, prior planning to allow data collection flexibility based on the changing pandemic restrictions was necessary. The COVID-19 restrictions created inaccessibility to some of the study participants, who would have preferred in-person interviews. According to a survey conducted by the United Nations Statistics Division and the World Bank in May 2020, 96 percent of national statistical offices partially or fully stopped in-person data collection [47]. In response to these requirements, many national statistical offices quickly turned to telephone or web interviews as an alternative data collection mode to maintain continuity in the production of key indicators and monitor the health and socioeconomic impact of the pandemic [47]. According to a report on the impact of the COVID-19 lockdown on early career researchers in the United Kingdom, more than 75% experienced a negative impact on their ability to continue with data collection, collaborate with colleagues, and disseminate research findings [48]. The government-enforced restrictions also had a negative impact on data analysis, writing, and working on grant or fellowship applications. More than one-third reported reduced access to the software needed for research, highlighting the significance of universities in providing better access to essential work resources. This resulted in increased work stress and uncertainty about the future. In our study, the option for in-person interviews was delayed until the pandemic restrictions were lifted, and participants and staff were more comfortable with this data collection modality.

Older adults are less likely than younger adults to use new technology [49]. This variation in technology use has the potential for unfavorable impact on research. In our study, low technological savviness, internet inaccessibility/ irregularities, and non-possession of telephones were some of the deterrents to the administration of online and telephone surveys. Some of the participants could only be reached through their relatives. The difference in technological usage between the younger and older adult populations is often due to age-related income disparities, perception of actual need to use the technology, products being too difficult to use, lack of knowledge, negative attitudes, and age-related changes such as vision and hearing loss, as well as fine motor challenges [49–51]. Results of a survey on the impact of technology on the daily lives of the older population in the Greater Cincinnati/Northern Kentucky area showed that they viewed technology positively and acknowledged the need for it. They agreed that using technology would produce a better quality of life for themselves and society [50]. However, although they were willing to learn technological skills, they had not taken steps toward the learning process. On the contrary, opposing reports showed that despite the marked and favorable advantages provided by technology, many older adults were reluctant to embrace it [51]. These individuals were portrayed as more conservative, traditional, skeptical, suspicious, cautious, and prudent when they encountered innovations. It is possible that there has been a shift toward favoring technology due to its extensive use during the pandemic, but more research is needed in this area. Methods utilized for research among older adults must be compatible with their lifestyle and values, suitable for their needs, and easily assimilated into their life. They must be easily understood, with clear evidence of benefits over the previous or traditional methods [49, 51]. Additionally, new methodologies should be piloted before use to enable older adults to gain more knowledge and provide feedback about usability and usefulness.

In addition to research methodology, research instruments used for older adults must be appropriate for their age and cultural background. While the DHQ III has been successfully utilized in several large studies, including the National Health and Nutrition Examination Survey (NHANES), it is long and detailed and may be viewed as burdensome for older adults. Our approach to administering it using an interview approach was well received, perceived as less burdensome by the participants, and reduced reporting errors. The YPAS questionnaire and food access questionnaires, also administered via interview, were less demanding. However, data collection volunteers reported feedback that these validated questionnaires included some items that were not common or lacked everyday relevant items. Researchers should consider customizing research instruments to their specific population.

## Conclusion

Despite challenges related to the COVID-19 pandemic, detailed data on the diet and exercise habits of older African American adults were obtained. Reflections on our study design, opportunities, challenges, and lessons learned will benefit other researchers during and post-pandemic. More importantly, forthcoming results from our study will inform interventions and policies aimed at minimizing current and future ramifications of poor diet and exercise habits during the pandemic in this vulnerable population.

## Figures and Tables

**Table 1:** Participant enrollment in Parent Study and C-FED Study

Study group	Recruitment status	No. of participants
Parent study*	Enrolled	133
C-FED study	Completed all surveys	118
	Completed physical activity/food security surveys only	5
	Declined participation	5
	Unable to contact	5
Total		133

* As of July 2022

**Table 2:** Characteristics of C-FED participants (*n* = 123)

Characteristic	*N* = 123	%
Age (years)		
55–59	21	17.1
60–64	19	15.5
65–69	32	26.0
70–74	24	19.5
75–79	14	11.4
≥ 80	13	10.6
Gender		
Male	37	30.1
Female	86	69.9
Education		
Less than high school	9	7.3
High school	31	25.2
Some college	48	39.0
Bachelor’s Degree	13	10.6
Master’s, Professional, or Doctoral Degree	14	11.4
Preferred not to respond/ others	8	6.5
Marital status		
Married or living with companion	44	38.3
Not currently married	71	61.7
Self-rated physical health		
Excellent or very good	33	27.5
Good	45	37.5
Fair or poor	42	35.0
COVID-19 vaccination status		
Not vaccinated	12	9.8
Partially vaccinated	7	5.7
Fully vaccinated	104	84.6
Select CVD comorbidities		
Hypertension (HTN)	70	58.3
Diabetes (DM)	28	23.1
BMI ≥ 30 (obese (OB))	52	45.2
BMI ≥ 25 (overweight or obese (O/O))	89	77.4
Frequency of select comorbidities above		
None	9	8.0
One	42	37.5
Two	48	42.9
All three	13	11.6
Number of prescribed medications		
0	69	56.1
1 or 2	32	26.0
3 or more	22	17.9
Insurance status		
Medicare	58	47.2
Medicaid	24	19.5
Private	36	29.3
VA	2	1.6
Uninsured	3	2.4

**Table 3:** Summary of opportunities and challenges associated with assessing food insecurity, exercise, and dietary history among older African American parishioners during the COVID-19 pandemic

Opportunities		
Opportunity	Identified benefits	Implemented strategy and/or lessons learned
Importance of study findings	Importance of study findings for future interventions on local food access and diet and exercise behaviors	*Study methodology *Resulting findings
Community-based participatory research (CBPR) approach	Partnership and rapport with community strengthened	*Goals based on community needs *Leveraging trusted church leaders *Appointment of a church leader as a paid community faculty and member of core study team *Partnership agreements
Dissemination of survey results and modeling	Knowledge translation and exchange	*Gala event with individualized report review *Healthy meal served to model healthy eating *Enhanced informal networking
Empowerment and longevity	Health ambassadors empowered for future health interventions	*Use of trusted peers as health ambassadors
Leveraging established research protocol	Resource and responsibility increased efficiency for participants and study staff	*Participant convenience e.g. combined consent form, survey completion in one sitting, study logistics etc *Shared responsibilities between Parent Study and C-FED study
Multiple data collection modalities	Flexibility/convenience increase participation	Participants selected survey modality: selfadministration, telephone interviews or in-person
Data storage	Cloud-based, eliminate data entry burden, transcription errors, and data loss	*Cloud-based data entry modalities identified early *Enhanced accessibility to data provided realtime monitoring of data collection feedback
Challenges		
Challenge	Identified barriers	Implemented strategy
COVID-19 restrictions	In person interviews not possible although preferred by some	*Flexible survey delivery modalities, including video calls *In-person interviews when some COVID-19 restrictions were lifted
Recruitment challenges	*Parent Study and C-Fed study misunderstanding by participants *Difficulty reaching consented participants	*Study information clarified often *Weekly meetings to obtain feedback, answer questions, and strengthen
Logistical challenges	*Delayed data collection	**Constant communication between Parent study and C-FED study teams
Participant technical challenges	*Limited technological savviness *Limited internet accessibility/reliability	*Use of trusted peers, such as ambassadors, church leaders, and relatives to contact participants for telephone interviews and online surveys *In-person interviews subsequently available *In-person hosting church provided a Hotspot *Church ambassadors provided laptops
Recall and study fatigue	*Older adults’ difficulty with recall *Long surveys	*Opportunity to complete surveys in two sessions
Interrater reliability	5 data collection volunteers	*Rigorous training of data collection volunteers *Biweekly data collection volunteer meetings *Random data checks, corrections, and feedback

## References

[1] Mackey K, Racial and ethnic disparities in COVID-19-Related infections, hospitalizations, and deaths : a systematic review. Ann Intern Med. 2021;174(3):362–73. 33253040 10.7326/M20-6306 PMC7772883

[2] Snowden LR, Graaf G. COVID-19, social determinants past, present, and future, and African Americans’ Health. J Racial and Ethnic Health Disparities. 2021;8:12–20. 10.1007/s40615-020-00923-3 PMC7682952 33230737

[3] CDC. Risk for COVID-19 infection, hospitalization, and death by race/ethnicity. http://www.cdc.gov/coronavirus/2019-ncov/covid-data/investigations-discovery/hospitalization-death-by-race-ethnicity.html. Accessed 10.5.2022.

[4] Tai DBG, The disproportionate impact of COVID-19 on racial and ethnic minorities in the United States. Clin Infect Dis. 2021;72(4):703–6. 32562416 10.1093/cid/ciaa815 PMC7337626

[5] Micha R, Association Between dietary factors and mortality from heart disease, stroke, and type 2 diabetes in the United States. JAMA. 2017;317(9):912–24. 28267855 10.1001/jama.2017.0947 PMC5852674

[6] XuF, Relationship between diet quality, physical activity and health-related quality of life in older adults: findings from 2007–2014 National Health and Nutrition Examination Survey. J Nutr Health Aging. 2018;22(9):1072–9.30379305 10.1007/s12603-018-1050-4

[7] Morris MC, Associations of vegetable and fruit consumption with age-related cognitive change. Neurology. 2006;67(8):1370–6. 17060562 10.1212/01.wnl.0000240224.38978.d8 PMC3393520

[8] Nicklett EJ, Kadell AR. Fruit and vegetable intake among older adults: a scoping review. Maturitas. 2013;75(4):305–12. 23769545 10.1016/j.maturitas.2013.05.005 PMC3713183

[9] Kwon SC, Physical activity, fruit and vegetable intake, and health-related quality of life among older Chinese, Hispanics, and Blacks in New York City. Am J Public Health. 2015;105(Suppl 3):S544–52. 25905844 10.2105/AJPH.2015.302653 PMC4455524

[10] Ervin RB. Healthy Eating Index scores among adults, 60 years of age and over, by sociodemographic and health characteristics: United States, 1999–2002. Adv Data. 2008;20(395):1–6. 18646580

[11] Vergis S Diet Quality and nutrient intake of urban overweight and obese primarily African American Older adults with osteoarthritis. Nutrients, 2018;10(4). 10.3390/nu10040485 PMC5946270 29652820

[12] Schering T, Association of diet quality and physical function among overweight and obese primarily African American older adults with lower extremity osteoarthritis. Nutr Healthy Aging. 2021;6(1):61–71. 33709042 10.3233/NHA-190081 PMC7903244

[13] Deierlein AL, Diet quality of urban older adults age 60 to 99 years: the cardiovascular health of seniors and built environment study. J Acad Nutr Diet. 2014;114(2):279–87. 24262516 10.1016/j.jand.2013.09.002 PMC3946974

[14] Hsiao PY, Dietary patterns and diet quality among diverse older adults: the University of Alabama at Birmingham Study of Aging. J Nutr Health Aging. 2013;17(1):19–25. 23299373 10.1007/s12603-012-0082-4 PMC3574872

[15] Nassim G, Nutrition self-efficacy and dietary patterns among older African American Women in Kansas. Kans J Med. 2020;13:209–13. 32843925 PMC7440852

[16] Hiza HAB, Diet quality of americans differs by age, sex, race/ethnicity, income, and education level. J Acad Nutr Diet. 2013;113(2):297–306. 23168270 10.1016/j.jand.2012.08.011

[17] Mattioli AV, Quarantine during COVID-19 outbreak: Changes in diet and physical activity increase the risk of cardiovascular disease. Nutr Metab Cardiovasc Dis. 2020;30(9):1409–17. 32571612 10.1016/j.numecd.2020.05.020 PMC7260516

[18] Elisabeth AL, Karlen SB, Magkos F. The effect of COVID-19-related lockdowns on diet and physical activity in older adults: a systematic review. Aging Dis. 2021;12(8):1935–47. 34881078 10.14336/AD.2021.0606 PMC8612611

[19] Harrison E, COVID-19 pandemic-related changes in wellness behavior among older Americans. BMC Public Health. 2021;21(1):755. 33874931 10.1186/s12889-021-10825-6 PMC8054850

[20] Nicklett EJ Food Access, diet quality, and nutritional status of older adults during COVID-19: a scoping review. Front Public Health. 2021;9. 10.3389/fpubh.2021.763994 PMC8669368 34917577

[21] Parekh N Food insecurity among households with children during the COVID-19 pandemic: results from a study among social media users across the United States. Nutr J. 2021;20(73). 10.1186/s12937-021-00732-2 PMC8403824 34461913

[22] Niles MT The Early Food Insecurity Impacts of COVID-19. Nutrients. 2020. 12(7). 10.3390/nu12072096 PMC7400862 32679788

[23] Morales DX, Morales SA, Beltran TF. Racial/ethnic disparities in household food insecurity during the COVID-19 Pandemic: a nationally representative study. J Racial Ethn Health Disparity. 2021;8:1300–14. 10.1007/s40615-020-00892-7 PMC7556612 33057998

[24] Choi SL, Men F. Food insecurity associated with higher COVID-19 infection in households with older adults. Public Health. 2021;200:7–14. 34653739 10.1016/j.puhe.2021.09.002 PMC8433037

[25] Dubowitz T, Food insecurity in a low-income, predominantly African American cohort following the COVID-19 pandemic. Am J Public Health. 2021;111(3):494–7. 33476228 10.2105/AJPH.2020.306041 PMC7893363

[26] Higashi RT, Experiences of increased food insecurity, economic and psychological distress during the COVID-19 pandemic among Supplemental Nutrition Assistance Program-enrolled food pantry clients. Public Health Nutr. 2022;25(4):1027–37. 34865672 10.1017/S1368980021004717 PMC8712963

[27] COVID-19: Keeping Los Angeles safe:summaries of data related to the COVID-19 response in Los Angeles. 2021. [cited 2021 10.01.2022]; Available from. https://coronavirus.lacity.org/data.

[28] BioPsychoSocial Assessment Tools for the Elderly-Assessment Summary Sheet. Yale Physical Activity Questionnaire (YPAS). 1988. Available from. https://instruct.uwo.ca/kinesiology/9641/Assessments/Biological/YPAS.html. Accessed 12.10.2022.

[29] De Abajo S, Larriba R, Marquez S. Validity and reliability of the Yale Physical Activity Survey in Spanish elderly. J Sports Med Phys Fitness. 2001;41(4):479–85. 11687767

[30] Young DR, Jee SH, Appel LJ. A comparison of the Yale Physical Activity Survey with other physical activity measures. Med Sci Sports Exerc. 2001;33(6):955–61. 11404661 10.1097/00005768-200106000-00015

[31] Diet History Questionnaire III (DHQ III). Available from. https://epi.grants.cancer.gov/dhq3. Accessed 10.5.2022.

[32] Subar AF, Comparative validation of the Block, Willett, and National Cancer Institute food frequency questionnaires : the Eating at America’s Table Study. Am J Epidemiol. 2001;154(12):1089–99. 11744511 10.1093/aje/154.12.1089

[33] Subar AF, Using intake biomarkers to evaluate the extent of dietary misreporting in a large sample of adults: the OPEN study. Am J Epidemiol. 2003;158(1):1–13. 12835280 10.1093/aje/kwg092

[34] USDA. MyPlate U.S. Department of Agriculture: older adults. Available from: https://www.myplate.gov/life-stages/older-adults. Accessed 12.10.2022.

[35] Campbell MK, Process evaluation of an effective church-based diet intervention: Body & Soul. Health Educ Behav. 2007;34(6):864–80. 17200096 10.1177/1090198106292020

[36] Hippolyte JM Recruitment and retention techniques for developing faith-based research partnerships, New York city, 2009–2012. Prev Chronic Dis. 2013. 10(E30). 10.5888/pcd10.120142 PMC3603629 23469766

[37] Yeary KH Feasibility of an evidence-based weight loss intervention for a faith-based, rural, African American population. Prev Chronic Dis. 2011. 8(6). PMC3221585 22005639

[38] Lona M, Recruitment and retention of older adults in aging research. J Am Geriatr Soc. 2008;56(12):2340–8. 19093934 10.1111/j.1532-5415.2008.02015.x PMC2695395

[39] Chelsie A Recruiting and engaging older men in evidence-based health promotion programs: perspectives on barriers and strategies. J Aging Res. 2016. pp 1–8. 10.1155/2016/8981435 PMC4913010 27366330

[40] Swann SA, Meaningful community collaboration in research. BC Med J. 2020;62(9):340–1.

[41] Mayo-Gamble TL A multiperspective on the broad dissemination of research findings to past research participants and the community-at-large. Transl Behav Med. 2022. 12(1). 10.1093/tbm/ibab095 34244808

[42] Schroter S, Frequency and format of clinical trial results dissemination to patients: a survey of authors of trials indexed in PubMed. BMJ Open. 2019;9(10):e032701. 10.1136/bmjopen-2019-032701 PMC6803145 31636111

[43] Mitchell J, Building and sustaining a community advisory board of african american older adults as the foundation for volunteer research recruitment and retention in health sciences. Ethn Dis. 2020;30(Suppl):755–64. 33250622 10.18865/ed.30.S2.755 PMC7683030

[44] Sivakumar SF, J. Collaborations: pros and cons. 2016. [10/10/2022]; Available from. https://www.ascb.org/careers/41032-2/.

[45] Forsat ND, Recruitment and retention of older people in clinical research: a systematic literature review. J Am Geriatr Soc. 2020;68(12):2955–63. 33075140 10.1111/jgs.16875

[46] Chen MS, Twenty years post-NIH Revitalization Act: enhancing minority participation in clinical trials (EMPaCT): laying the groundwork for improving minority clinical trial accrual. Cancer. 2014;120:1091–6. 24643646 10.1002/cncr.28575 PMC3980490

[47] Planning and Implementing Household Surveys Under COVID-19: Technical Guidance Note. 2020 07.07.2022]; Available from. https://unstats.un.org/iswghs/news/docs/COVID-19_TechnicalGNote_final.pdf.

[48] Byrom N The challenges of lockdown for early-career researchers. eLife. 2020. 9. 10.7554/eLife.59634 PMC7292644 32530421

[49] Dan F, Designing for older adults. London: CRC Press; 2014.

[50] Li YB, Perkins A. The impact of technological developments on the daily life of the elderly. Technol Soc SciDirect. 2007;29(3):361–8.

[51] Wu YH, Bridging the digital divide in older adults: a study from an initiative to inform older adults about new technologies. Clin Interv Aging. 2015;10:193–200. 25624752 10.2147/CIA.S72399 PMC4296914

[52] Los Angeles County Regions map. 2022. Available from: https://www.alamy.com/stock-photo-los-angeles-county-regions-map-121497196.html.

